# Adalimumab-Induced Erythema Multiforme in a Patient With Rheumatoid Arthritis: A Case Report

**DOI:** 10.7759/cureus.21126

**Published:** 2022-01-11

**Authors:** Jinan Q Mohammed, Zainab Mahmmod, Abdulsatar J Mathkhor

**Affiliations:** 1 Dermatology, Basrah Teaching Hospital, Basrah, IRQ; 2 Department of Medicine, College of Medicine/ University of Basrah, Basrah, IRQ; 3 Rheumatology, Basrah Teaching Hospital, Basrah, IRQ

**Keywords:** rheumatoid arthritis, erythema multiforme, case report, anti-tumor necrosis factor-alpha, adalimumab

## Abstract

Tumor necrosis factor-alpha (TNF-α) inhibitors are a group of biological medications that revolutionized the treatment of rheumatoid arthritis (RA) and several other inflammatory autoimmune diseases. The wide use of these drugs has been associated with some adverse reactions. Erythema multiforme (EM) is an immune-mediated cutaneous disorder that represents a hypersensitivity reaction to infections, vaccines, and some medications. We present a female patient with EM with characteristic skin lesions following anti-TNF-α medication adalimumab for rheumatoid arthritis.

## Introduction

Treatment of RA has been revolutionized by discovering the role of certain cytokines, particularly tumor necrosis factor-alpha (TNF-α), in the pathogenesis of the disease. TNF-α plays a pivotal role in the inflammatory and immune response, with powerful effects on many aspects of cellular and humoral immunity [1]. The approach of targeting TNF-α has been significantly associated with the improvement of RA. Five different anti-TNF-α drugs have entered clinical use: infliximab (IFX), adalimumab (ADA), etanercept (ETC), golimumab (GLM), and certolizumab pegol (CZP) have been used for the treatment of RA [2]. Though different studies have successfully demonstrated the efficacy and safety of anti-TNF-α, there is still debate regarding the potential untoward effects of these biologicals [3]. Serious adverse reactions involving the skin and oral mucosa have been reported with the use of the fully-humanized monoclonal antibody adalimumab. These adverse reactions are hypersensitivity reactions, a lupus-like reaction, demyelinating disease, Stevens-Johnson syndrome (SJS), and Erythema Multiforme (EM) [4,5]. EM is an acute, self-limiting immune-mediated cutaneous disorder typically associated with hypersensitivity reactions to viruses primarily of the herpes simplex virus [6]. It is clinically characterized by targetoid lesions symmetrically distributed on the extremities with a lack of or minor mucosal involvement (minor form) or significantly affecting one or more mucous membranes (major form) [7]. There are scanty publications in the literature that reported the association of EM with anti-TNF-α. EM has been reported with infliximab, golimumab, etanercept, and adalimumab [8-10]. This report presents a woman who developed cutaneous lesions consistent with EM following treatment with adalimumab.

## Case presentation

A 58-year-old female patient visited our biologic therapy center with non-pruritic skin lesions that affected her extremities. The patient presented with a 20-year-history of RA. She was treated with parenteral methotrexate (15 mg/week), folic acid (5mg/week), prednisolone (5mg/day), and omeprazole (40 mg/day), and she was in clinical remission for the last several years. Two years ago, she described morning stiffness of more than one hour, joint pain, and swelling with high disease activity. Etanercept (50mg/week) was added to her treatment regimen, and clinical remission was achieved. Five months ago, secondary failure of etanercept was noticed. The patient deteriorated, with severe joint pain, swelling and stiffness. On physical examination, her wrists, metacarpophalangeal (MCP) joints of both hands, both knees revealed swelling, tenderness, and increased heat with limited range of movement. Laboratory findings: erythrocyte sedimentation rate, C-reactive protein, and Leukocytes were increased, while hemoglobin was decreased (Table 1).

**Table 1:** Laboratory findings

Investigation	Value	Normal range
Erythrocyte sedimentation rate	70 mm/h	< 20 mm/h
C-reactive protein	17 mg/L	< 10 mg/L
Leukocytes	11900/μL	4000- 11000/μL
Hemoglobin	8.7 g/dl	12- 16 g/dl

Therefore, adalimumab, another anti-TNF-α, was administered instead of etanercept (40mg/ eow), and the patient achieved clinical remission after the 3rd dose. At that time, the patient developed non-pruritic skin lesions that affected her extremities. On examination, there were targetoid papules and plaques on her forearms, elbows, knees, and wrists, trunk, and the mucosa was spared. There were multiple lesions with a diameter of 1-2 cm and a typical targetoid appearance with a central dark red zone covered by crust, an intermediate pink and edematous zone with external erythema. The patient had no signs of active infection or consumed any medicine apart from her treatment regimen prior to the occurrence of the skin lesions. A skin biopsy was obtained from the lesion and revealed EM. Histopathologic examination revealed a vacuolar interface dermatitis with a dense, mainly lymphocytic infiltrate with many eosinophils. Adalimumab was discontinued, an oral antihistamine, local antiseptic, and corticosteroid were applied to the lesion.

**Figure 1:**
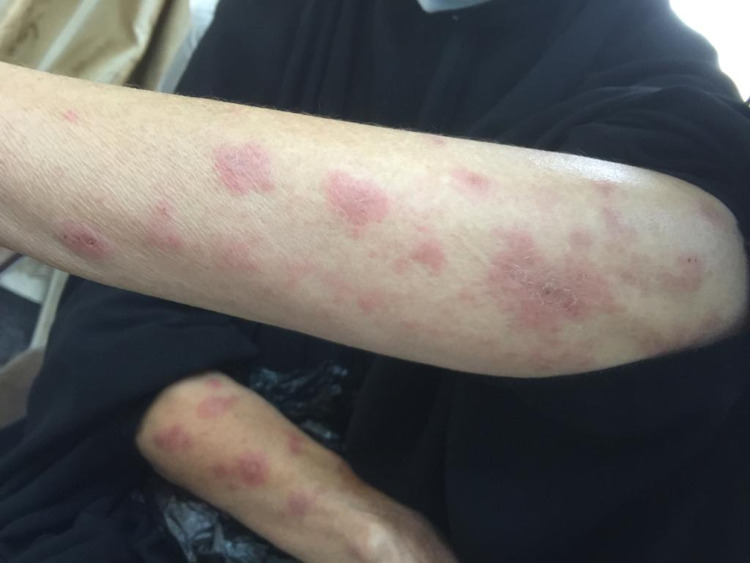
Cutaneous presentation of multiple erythematous papules with a central dusky appearance on both forearms.

## Discussion

Since the development of anti-TNF and the increase in its uses for different autoimmune inflammatory rheumatic diseases, many side effects have been reported. One of these side effects is cutaneous manifestations. Injection site reactions may occur with the use of different anti-TNF medications such as etanercept, adalimumab, and certolizumab pegol. These reactions clinically present as redness, itching, pain, swelling, bruising, and/or irritation at the injection site. An allergic hypersensitivity reaction and local trauma are the most important mechanisms for injection reactions [4]. Infusion reactions may occur with infliximab (IFX) that are classified as acute or delayed, and both are associated with cutaneous manifestations [11]. Serious cutaneous adverse reactions have been reported during therapy with TNF inhibitors adalimumab, etanercept, and infliximab, including EM and Stevens-Johnson syndrome [12]. The occurrence of EM in patients treated with diverse TNF inhibitors suggests that EM is a cutaneous side-effect of this class of biologic agents. A humanized antibody against TNF$\alpha$ or adalimumab may potentially cause less immune-mediated skin reactions in comparison to infliximab. In this case, cutaneous reactions occurred after using adalimumab. These targetoid lesions were confirmed to be erythema multiforme by the histopathological examination of skin biopsy. There was no other reported cause for the development of this lesion rather than using this anti-TNF; therefore, adalimumab was the implicated cause in this case. The important point that confirms the causal relationship between adalimumab and the development of EM is the disappearance of rash after the discontinuation of the drug. The occurrence of EM in patients treated with diverse TNF inhibitors suggests that EM is a cutaneous side-effect of this class of biologic agents [10].

## Conclusions

EM is an immune-mediated cutaneous disorder that can occur as a hypersensitivity reaction to different causative agents. The occurrence of EM in patients treated with adalimumab with no other obvious cause suggests that EM is a cutaneous side-effect of this class of biological agents.
